# Some Clinical Aspects of Dysmenorrhœa
1A Paper read at a Meeting of the Bristol Medico-Chirurgical Society on April 13th, 1927.


**Published:** 1927

**Authors:** R. S. S. Statham

**Affiliations:** Honorary Gynæcologist, Bristol Royal Infirmary; Lecturer on Obstetrics in the University of Bristol


### SOME CLINICAL ASPECTS OF DYSMENORRHŒA.[1]

BY

R. S. S. Statham, O.B.E., M.D., Ch.M.,

*Honorary Gynæcologist, Bristol Royal Infirmary; Lecturer on Obstetrics in the University of Bristol.*

---

It is probable that dysmenorrhœa, in a more or less degree, is one of the very commonest of the physiological errors which occur in modern civilisation. It is most unusual to find menstruation occurring without some degree of pain or unaccompanied by unpleasant molimina. I, personally, know of two cases only, and they regard themselves as unusually fortunate.

This condition is intimately connected with modern civilisation. Cameron points out that "in large cities" 30 per cent. of women suffer considerable discomfort during the menstrual period, and a very practical reason has been advanced by Professor Tuffier. He states that menstruation is not a normal function at all. In a savage and truly natural state this event must be a rarity in any individual. The woman marries very young, sees perhaps one or two periods, and then becomes pregnant. The next twelve to fifteen months are occupied in pregnancy and lactation, and then possibly one more period occurs before the next pregnancy, so that—as the menopause comes on

[1] A Paper read at a Meeting of the Bristol Medico-Chirurgical Society on April 13th, 1927.

early—the woman only menstruates a dozen times or so in her life. In modern civilised Europe the case is entirely different.

Before discussing dysmenorrhœa, it is necessary to remember the normal mechanism of menstruation. During the last few years a great amount of research has been undertaken on the menstrual function. Shaw, Marshall, and Beckwith Whitehouse, Novak and Schrœder have published many papers, and recently Beckwith Whitehouse has authoritatively summarised the latest views. He states that menstruation is the abortion of the decidua of the non-pregnant uterus; the menstrual flow corresponds with the failing of the corpus luteum when the ovum is not fertilised, and is the "lochia" of a menstrual abortion. This view is now universally accepted, I think, and this is in keeping with the French view that I have quoted, for surely a decidual abortion cannot be regarded as normal. With this conception of menstruation, it is hardly surprising that it should be so frequently complicated by pain and difficulty.

There are three main types of dysmenorrhœa described, and I wish to draw attention to a fourth type which I find to be very common.

These are:—

1. The spasmodic or intrinsic type of Blair Bell.
2. The congestive or extrinsic type of Blair Bell.
3. The membranous type.
4. The "mixed" type.

The proper recognition of each type makes all the difference to the principles of the treatment required.

1. *Spasmodic Dysmenorrhœa* is easily diagnosed as a rule. The patient gives a history that the pain began with the first period and has been present at every period since. The pain is acute and colicky in type, sometimes so severe that the patient vomits or even faints. The pain begins before the flow, and is usually much better when this has been established twenty-four hours or more. The loss is almost always scanty and irregular, and between the periods there is usually no discomfort.

If the patient be examined, in most cases a small, ill-developed uterus will be found. Frequently it is "cochleate," *i.e.* acutely anti-flexed, and may also be retroverted; in the angle made by the cervix and body irregular masses of muscle tissue are developed, and Cameron lays much stress on the consequent spasm of the internal os. In these cases the pain is not due to "pinhole os." Blood can pass up a very small capillary tube, and could certainly get out of a cervix which will admit a sound. The pain is due to irregular contraction of a malformed uterus, *i.e.* to muscular cramp, and is truly "intrinsic."

2. In the second, the *congestive or extrinsic* type, on the other hand, the history is that of a girl having normal periods for some time, when they become painful. The pain is dull and aching, the flow profuse, the pain is localised to the back and "drags down" into the pelvis. Between these periods the pain is constantly present, though to a less degree. On examination it is usual to find the uterus large and often fixed in a position of retroversion. The fornices are tender and often the ovaries and tubes are enlarged and palpable; in fact, there is a typical pelvic peritonitis. Further inquiry will elicit the fact that about 50 per cent. of these cases follow a confinement or miscarriage, and, in many of the remainder, appendicitis or an ascending infection will be described as a precursor of the trouble. Here the pain is due to the surrounding organs being adherent and inflamed, a condition made much worse by the normal pelvic congestion of menstruation.

3. *Membranous dysmenorrhœa* is a very rare condition. Personally I have only seen one case, in which the patient passed a large bolus-like cast of the endometrium. I imagine the pain was really due to obstruction, similar to that occurring when a polypus is extruded through the os. The cause is probably due to a failure in the ferment which normally causes the break up of the endometrium.

4. *The mixed case* is, to my mind, very common. There is nothing to prevent a girl with true intrinsic spasmodic dysmenorrhœa becoming infected with the gonococcus or getting an attack of appendicitis. I have operated on many such patients from whom my surgical colleagues have removed a suppurating appendix a year or so previously, and it has been the supervention of the extrinsic on the already existing intrinsic pain that has induced them to undergo a further operation. The importance of this will be seen when the question of treatment is considered.

### TREATMENT.

It is probable that greater strides have been made in the treatment of this condition than in any other gynæcological disease. The best indexes of this are the statements of medical officers in charge of large girls' schools. I was told by one such doctor that twenty years ago 75 per cent. of the girls had two or three days off work every month, and now only 10 per cent. were so excused. This is surely a remarkable improvement. The treatment must be considered under each type of case.

*Spasmodic Dysmenorrhœa.*—In this condition an enormous amount of good can be done by moral suasion. The girl must be encouraged not to consider herself an invalid. Exercise, the quieter games, and—above all—hot baths, will, in most cases, enable her to carry on her normal school life, and frequently the symptoms improve out of all knowing. This is due, I think, partly to development and education of the faulty uterus, and partly to the training in disregarding an unpleasant symptom. The latter factor is probably more powerful than is imagined, and an eminent psychiatrist told me that all cases of spasmodic dysmenorrhœa could be cured by psycho-analysis. This seems to me to be rather a strong claim, but I am sure that it is well founded. Should the pain be really severe, it will be found that rest in bed is very little good, but is convenient in view of the mental disturbance caused in a very young patient. The coal tar and allied products, *e.g.* aspirin, ammonal, etc., are a great help, and I have had several great successes with benzyl benzoate 20 per cent. in alcohol, combined with small doses of chloretone to prevent vomiting. It is unnecessary to state that morphia is more than dangerous for this type of patient. When I was house physician at Bethlem Royal Hospital I saw numbers of patients who had become addicts from being given morphine or heroine for this condition. If these methods fail, I do not think it wise to leave the patient too long before resorting to operation. The effect of severe and inevitable pain is very great in a child of 15 or 16, and she dreads its monthly recurrence more and more, and may become completely neurotic on this point. In 75 per cent. of cases a complete cure can be obtained by dilating the cervix to 14 Hegar. This simple operation has fallen into disrepute, and I am certain that the cause is that it has been done so often for the wrong type of dysmenorrhœa. It is quite useless to dilate the cervix if the patient is suffering from a chronic appendicitis or salpingitis, yet every day one sees cases of extrinsic dysmenorrhœa which have been so treated, and I am often told that "dilating has done no good" in a case where it was never indicated. In a true spasmodic dysmenorrhœa the passage of dilators straightens out the anti-flexion and ruptures the abnormal muscle masses about the internal os and produces instant and usually lasting relief. It is most important that the dilating be done slowly and carefully to avoid lacerations, or, as pointed out by Dr. Drew Smythe, the resulting scar tissue will cause a recurrence of the pain in an aggravated form. In all these cases I administer thyroid gland in hope that it will aid the uterus to develop to a proper extent, but I find it hard to say if it is very efficacious.

If this treatment is useless, it is difficult to know what to do. I have once done a subtotal hysterectomy in an unmarried woman of 31, whose life was a misery to her. She is now much improved, and very grateful, but I think it is a barely justifiable operation, and only to be resorted to in extreme cases, in women who are over 30 and unlikely to marry. Two other methods are described and very good results claimed, viz. sympathectomy of the plexus lying on the sheath of the internal iliac artery, and section of the "pelvic nerve" on the fourth lumbar vertebra. I have no personal experience of either method of treatment, but the results claimed are encouraging.

The *extrinsic cases* need quite a different line of treatment. Here the cause must be found and dealt with. The majority of cases do very well on aperients, *really* hot douching carried on for a quarter of an hour at least, and rest in bed during the flow. I do not find tampons much use, and they often cause pain by pressing on the swollen pelvic organs. Hot antiphlogistine tampons are useful, but require a skilled nurse to insert them. Usually a few weeks' treatment on these lines will improve the patient so much that she will not worry about her dysmenorrhœa, but if she does not get better fairly soon, it is necessary to open the abdomen and deal with the pelvic adhesions and displacements which are the root of the evil. I believe the appendix to be the most frequent cause of this type of dysmenorrhœa, and in a series of 150 operations for pelvic peritonitis I found it more or less involved in 82 cases.

It is obvious that in the "*mixed type*" of dysmenorrhœa both operations are necessary if the conditions are bad enough to warrant operation at all. It is most disappointing to open an abdomen and have the patient return in five or six months and tell one that the backache is gone, but that she still gets the colic or *vice versa.* I now do both operations at one sitting, and the dilating must be done first. If a Gilliam or similar ventrosuspension is performed, it is almost impossible to pull down the cervix and dilate it efficiently afterwards. This, of course, entails change of gown and gloves during the operation, but it is well worth it in results.

For the *membranous dysmenorrhœa* no efficient treatment has been devised. Curettage is recommended by some and gland therapy by others. My own experience of one case is quite useless to me to help form an opinion. She did not improve on any treatment at all, and finally had her uterus removed by somebody else.

There is one aspect of this complaint that I should like to stress, namely the great importance of spasmodic dysmenorrhœa about the time of the menopause, when it has not been present before. It is frequently the earliest sign of carcinoma of the body of the uterus, that organ trying to expel the growing mass in its interior, and so causing the colic. It is also the typical symptom of that interesting tumour, the endometrioma or adenomyoma. Here endometrial grafts or rests in the ovaries or other pelvic organs are found to contain hollow cavities into which menstrual blood is effused when the uterus menstruates. This blood being under great tension causes severe colicky dysmenorrhœa. It often occurs about the menopause, and I have twice mistaken the condition for a tubal pregnancy. The onset of spasmodic dysmenorrhœa in a woman of about 40 indicates an immediate curetting, and I have never regretted so doing, for a number of early carcinomata of the body have been found, and removed with every prospect of a cure.

